# EPIDEMIA - An EcoHealth Informatics System for integrated forecasting of malaria epidemics

**DOI:** 10.1186/1475-2875-13-S1-P92

**Published:** 2014-09-22

**Authors:** Michael Wimberly, Estifanos Bayabil, Belay Beyene, Geoffrey Henebry, Yi Liu, Abere Mihretie, Gabriel Senay

**Affiliations:** 1 South Dakota State University, Brookings, SD, USA; 2 Health, Development, and Anti-Malaria Association, Addis Ababa, Ethiopia; 3 Amhara Regional Health Bureau, Bahir Dar, Ethiopia; 4 USGS Earth Resources Observation and Science Center, Sioux Falls, SD, USA

## 

Advance information about the timing and locations of malaria epidemics can facilitate the targeting of resources for prevention and emergency response. However, predictions must be accurate to ensure that potential outbreaks are not missed and resources are not wasted responding to predicted outbreaks that do not occur. Early warning systems based on environmental monitoring can identify critical risk factors before an epidemic actually starts, but their accuracy is constrained by the influences of non-climatic factors on malaria transmission. In contrast, early detection of malaria epidemics based on epidemiological surveillance can be more reliable because it is conditioned on direct observations, but offers limited lead time and requires timely and accurate surveillance data. Ideally, a malaria forecasting system that combines elements of environmental monitoring and malaria case surveillance would be able to leverage the strengths and minimize the limitations of each approach. However, the potential for integration has been hindered by a dearth of established techniques and available tools for assimilating multiple data streams from malaria surveillance and environmental monitoring. In response to this need, we are developing the Epidemic Prognosis Incorporating Disease and Environmental Monitoring for Integrated Assessment (EPIDEMIA) computer system. EPIDEMIA will incorporate software for integrating and processing environmental and epidemiological data from multiple sources, data assimilation techniques that continually update models and forecasts, and a web-based interface that will make the resulting information useful to public health decision makers. The system will be tested by implementing it in the Amhara Region of Ethiopia in collaboration with local stakeholders in the NGO and Public Health sectors. We have conducted an initial co-design workshop in July 2014, with a requirements analysis that identified the main use cases for the public health community and the data requirements for the modelers. Future work will involve a cycle of implementation, training, usability testing, and continuous system development. This innovative translational bioinformatics approach will allow us to assess the practical effectiveness of the tools and continually upgrade and improve the technologies.

